# Supplementary material for: Pantothenate and L-Carnitine Supplementation Improves Pathological Alterations in Cellular Models of KAT6A Syndrome
Source: Genes (Basel). 2022 Dec 6;13(12):2300. doi: 10.3390/genes13122300 (PMC9778406; doi:10.3390/genes13122300)
Supplement: Supplementary file 1 [file genes-13-02300-s001.zip › genes-1941390-supplementary.pdf]

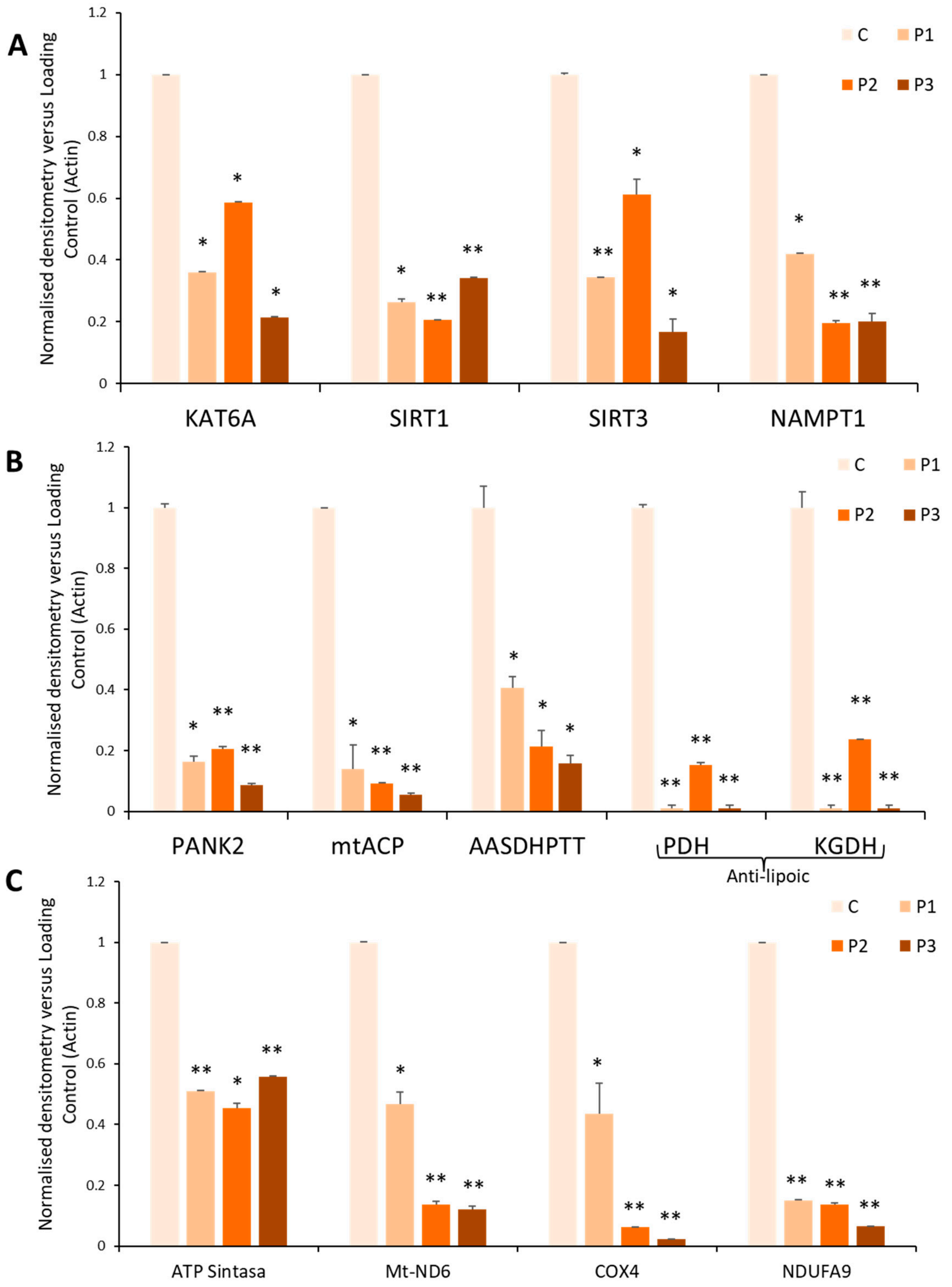

Supplementary Figure S1. Western blotting quantification of figure 1. Band densitometry of western blot shown in figure 1. Pathways in figure panels: acetylation-deacetylation (A), proteins related to CoA metabolism (B) and mitochondrial proteins (C). Data were normalized to actin. Data represent the mean ±SD of three separate experiments. *p-value < 0.05 and **p-value < 0.01.

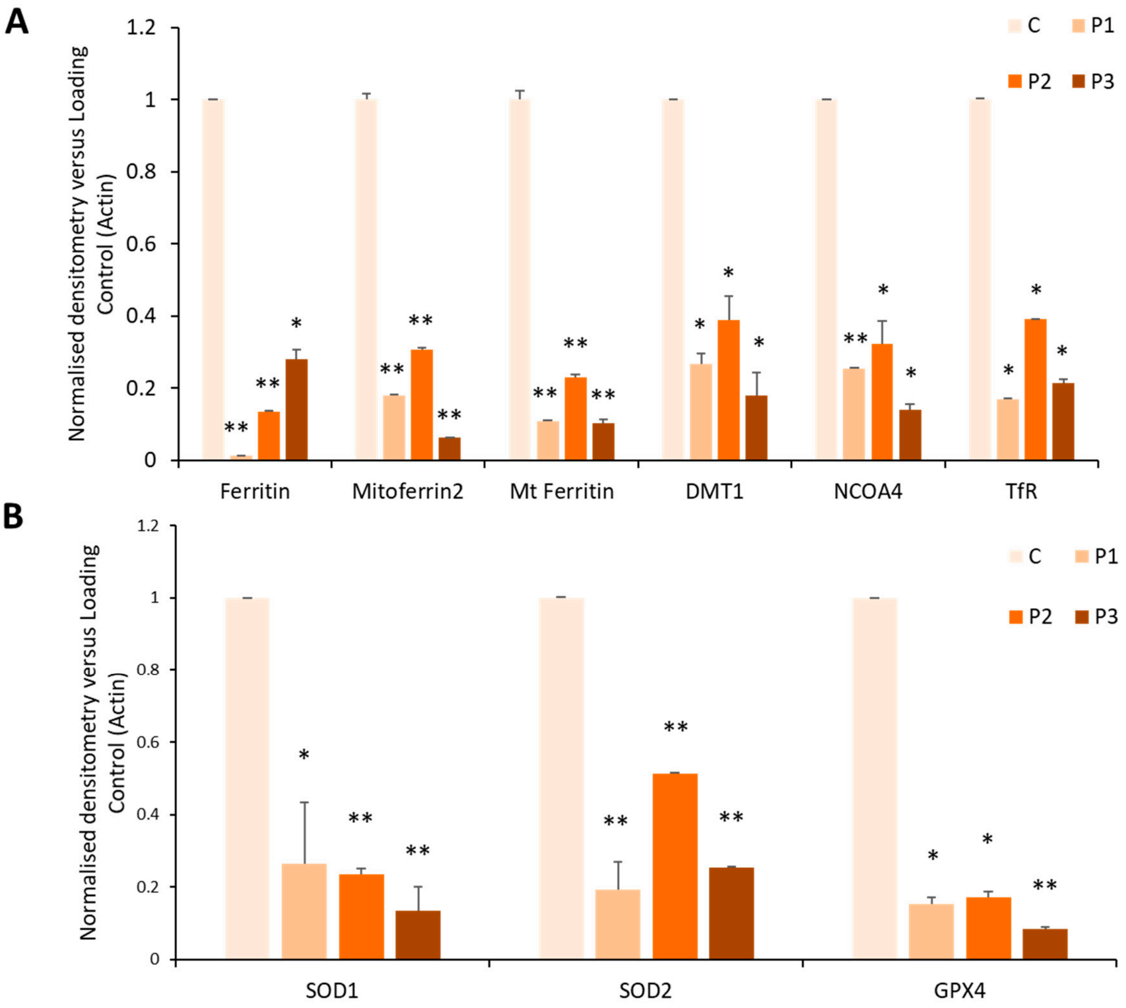

Supplementary Figure S2. Western blotting quantification of figure 1. Pathways in figure panels: proteins related to iron metabolism (A) and antioxidant enzymes (B). Data were normalized to actin. Data represent the mean ±SD of three separate experiments. *p-value < 0.05 and **p-value < 0.01.

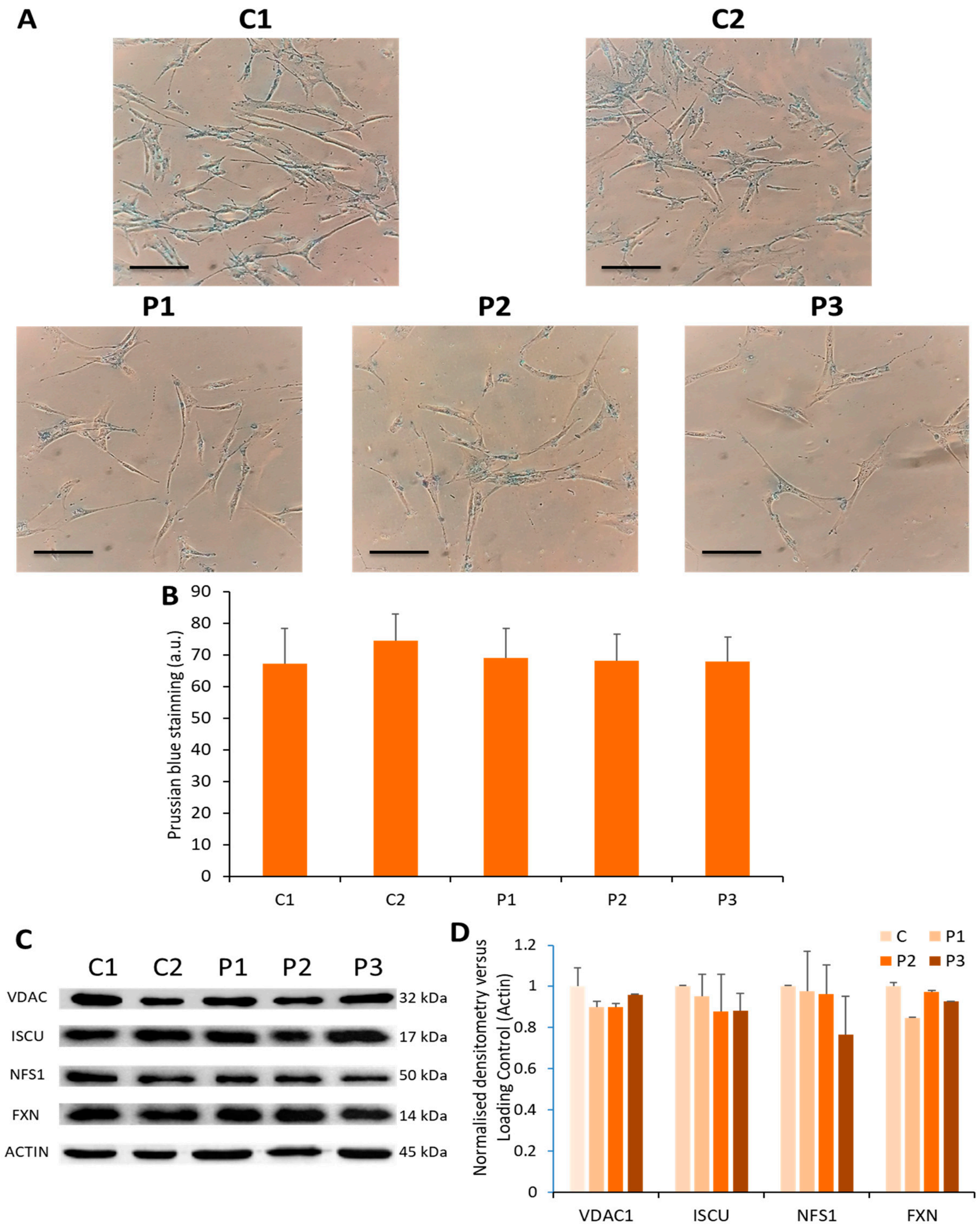

Supplementary Figure S3. Iron accumulation, mitochondrial mass and protein expression of iron-sulfur cluster biosynthesis. Iron accumulation in control (C1 and C2) and mutant KAT6A fibroblasts (P1, P2 and P3) assessed by Prussian Blue staining (A). Quantification of Prussian Blue staining of controls and patient fibroblasts (B). Protein expression patterns in control and KAT6A mutant fibroblasts. Protein extracts of Control (C1 and C2) and patient (P1, P2 and P3) were separated on a SDS polyacrylamide gel and immunostained with primary antibodies. (C) Proteins related to mitochondrial mass and iron-sulfur clusters biosynthesis: VDAC, ISCU, NFS1 and FXN. (D) Western blotting quantification of VDAC, ISCU, NFS1 and FXN. Data represent the mean ±SD of three separate experiments. Scale bar= 100 µm.

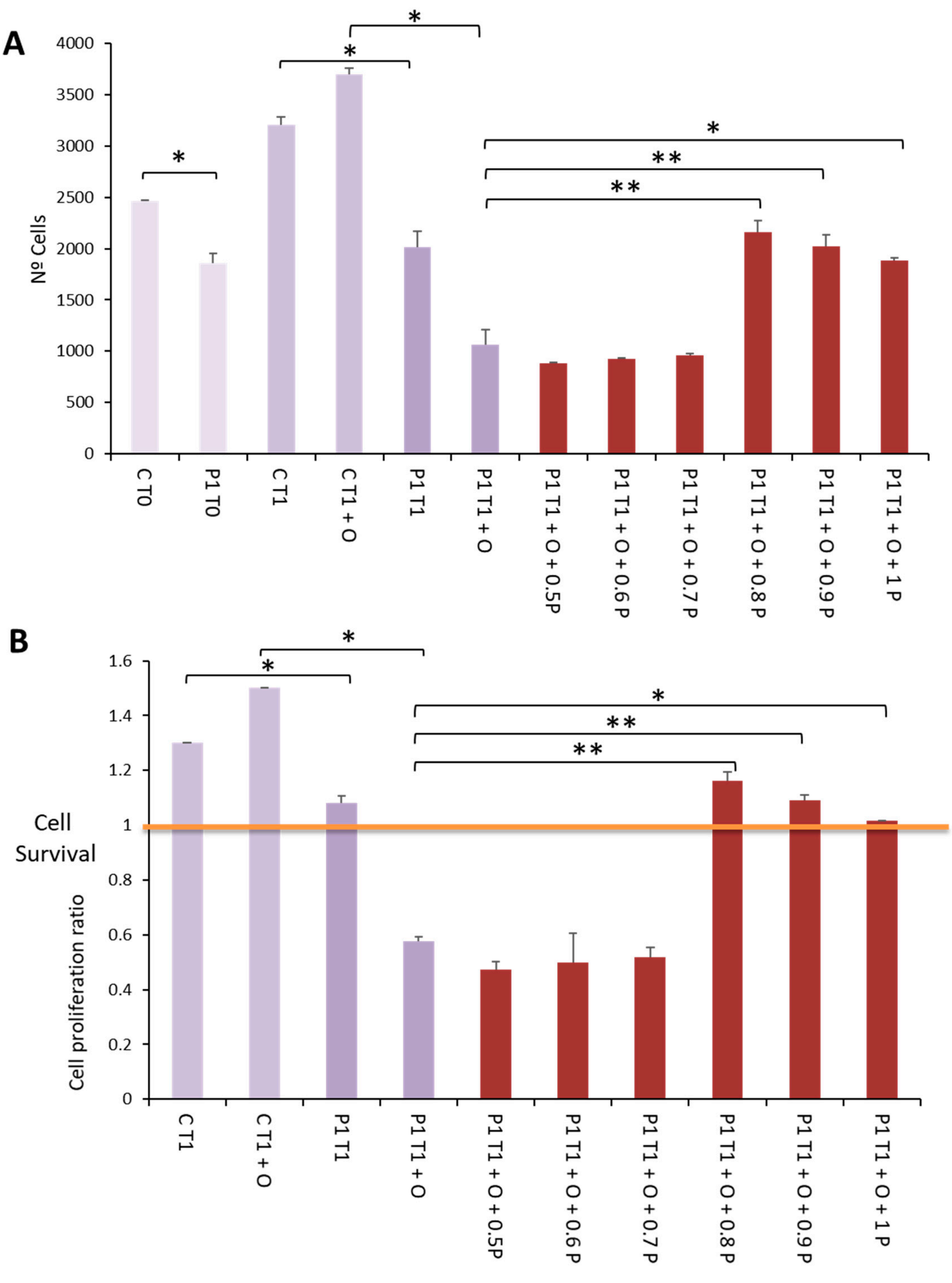

Supplementary Figure S4. Quantification of figure 2. (A) Cell proliferation rate of control (C) and mutant KAT6A (P1) fibroblasts in galactose medium with oligomycin at 0.5 nM (O). (B) Cell proliferation rate obtained from Nº Cells at T72h (T1)/ Nº cells at T0h (T0). Results ≥ 1 indicate cell survival and cell proliferation, results < 1 indicate cell death. The data represents the mean ±SD of 3 independent experiments. Pantothenate (P) was used at different concentrations (0.5-1 µM). Data represent the mean ±SD of three separate experiments. *p-value < 0.05 and **p-value < 0.01.

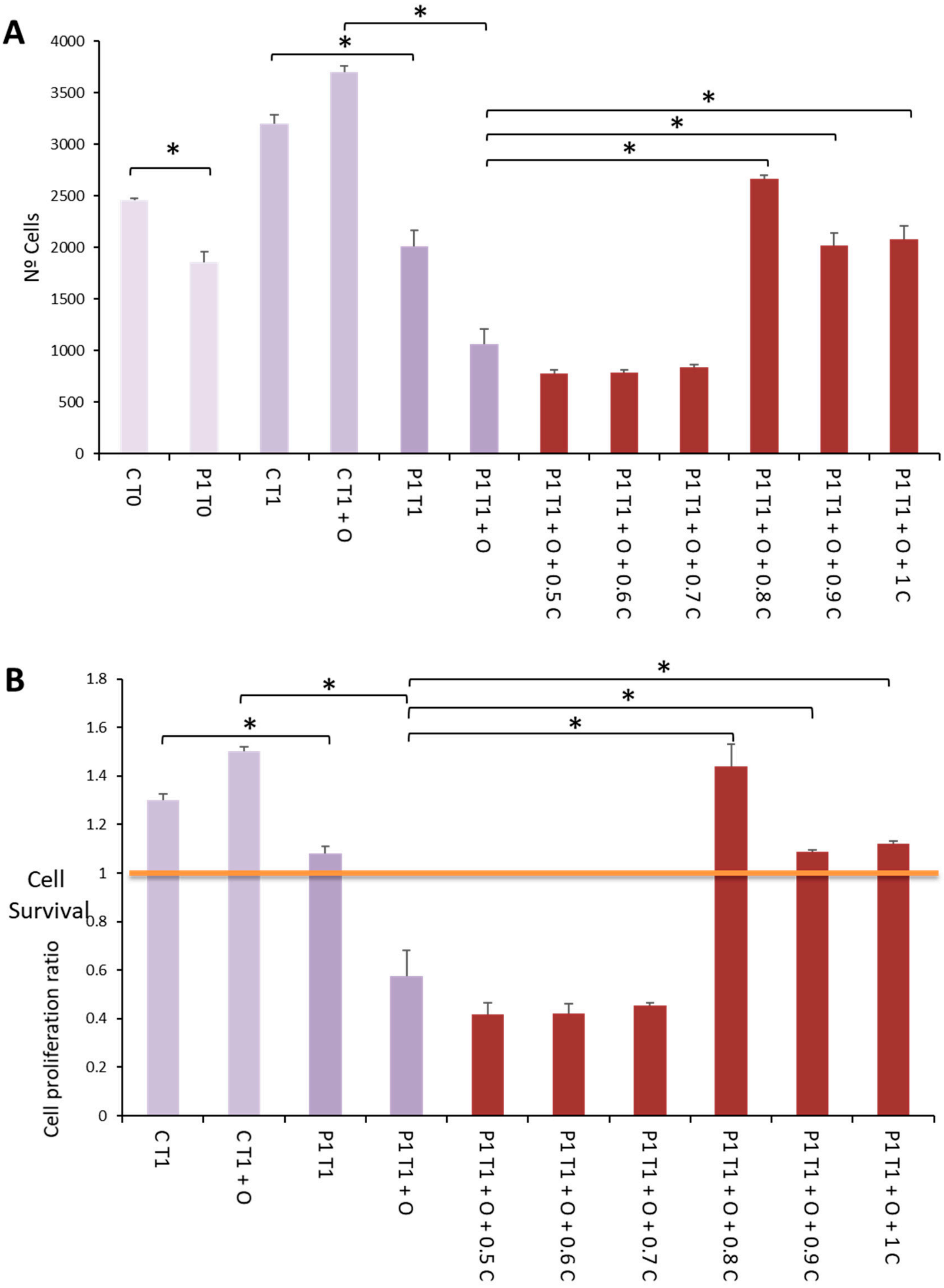

Supplementary Figure S5. **Quantification of figure 2.** (A) Cell proliferation rate of control (C) and mutant KAT6A fibroblasts (P1) in galactose medium with oligomycin at 0,5 nM (O). (A) Cell proliferation rate was obtained by the equation: Nº Cells at T72h (T1)/ Nº cells at T0h (T0). (B) Results ≥ 1 indicate cell survival and proliferation. Results < 1 indicate cell death. The data represents the mean ±SD of 3 independent experiments. L-carnitine (C) was used at different concentrations (0.5-1 µM). Data represent the mean ±SD of three separate experiments. *p-value < 0.05 and **p-value < 0.01.

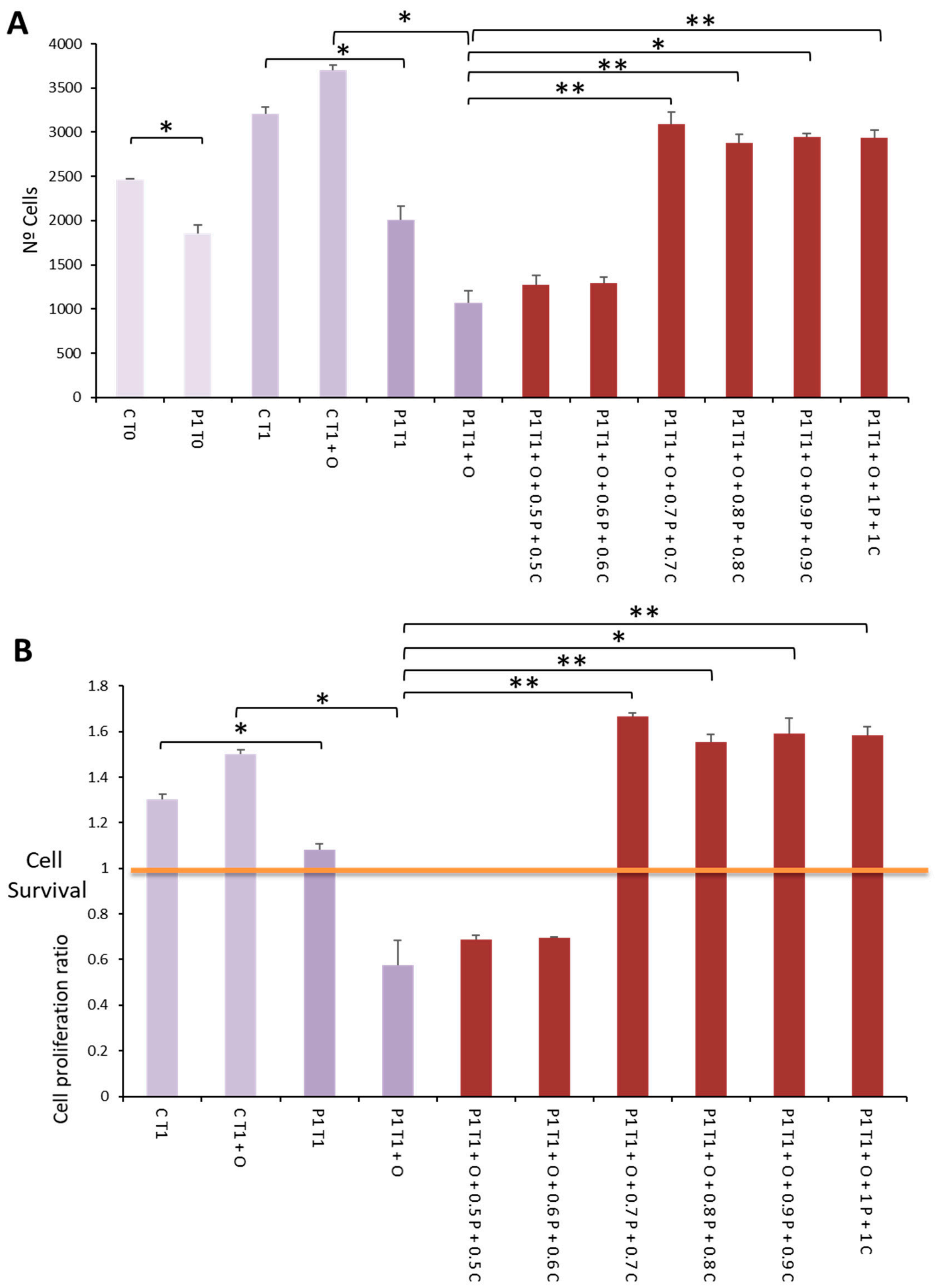

Supplementary Figure S6. Quantification of figure 2. (A) Cell proliferation rate of control (C) and mutant KAT6A fibroblasts (P1) in galactose medium with oligomycin at 0.5 nM (O). (B) Cell proliferation rate was obtained by the equation: Nº Cells at T72h (T1)/ Nº cells at T0h (T0). Results ≥ 1 indicate cell survival and proliferation. Results < 1 indicate cell death. The data represents the mean± SD of 3 independent experiments. The combination of pantothenate (P) and L-carnitine (C) was used at different concentrations (0.5-1 μM). Data represent the mean ±SD of three separate experiments. *p-value < 0.05 and **p-value < 0.01.

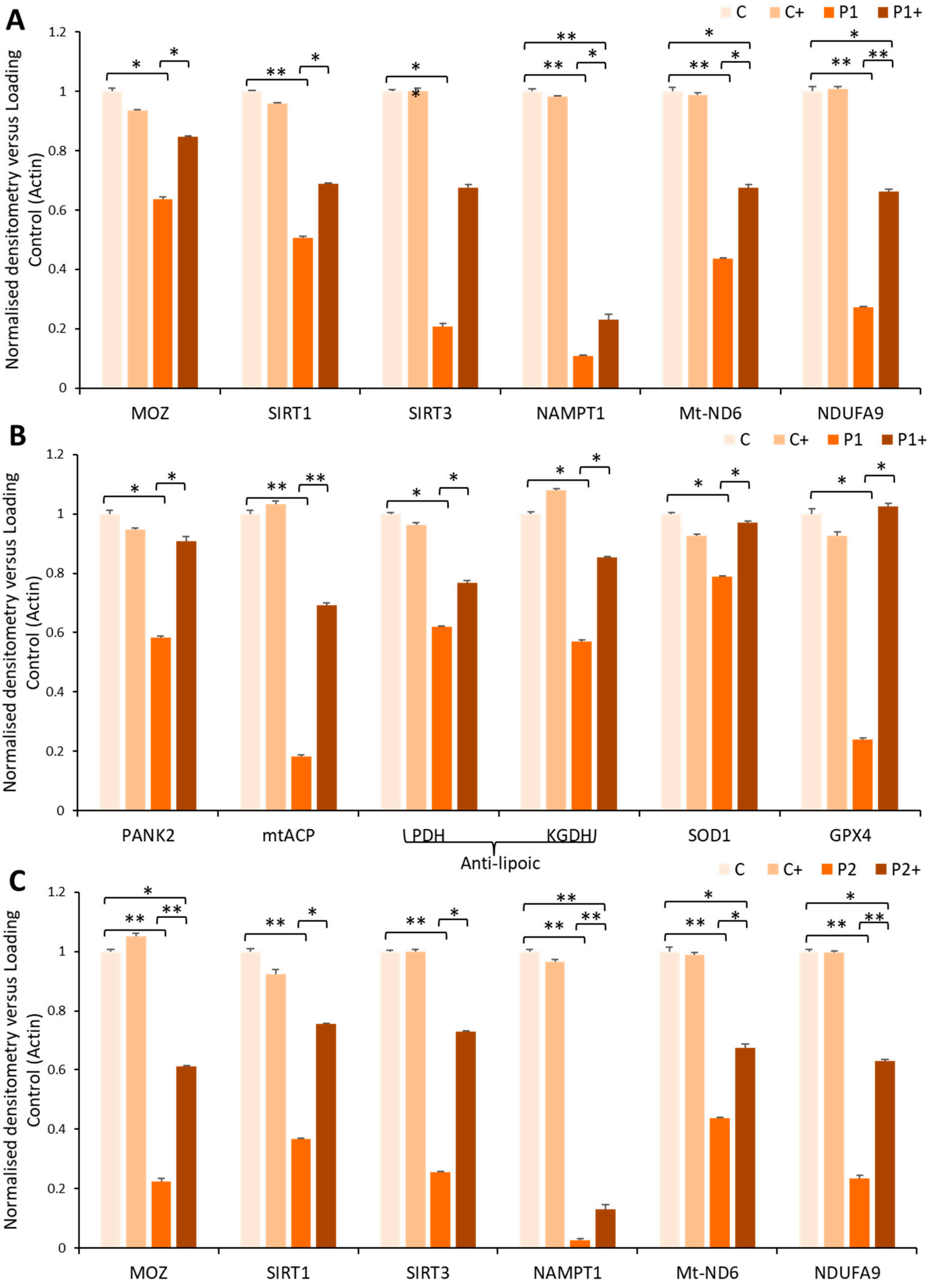

Supplementary Figure S7. Western blotting quantification of figure 3. Patients in figure panels: patient 1 (P1) (A-B) and patient 2 (P2) (C). Data were normalized to actin. Data represent the mean ±SD of three separate experiments. *p-value < 0.05 and **p-value < 0.01.

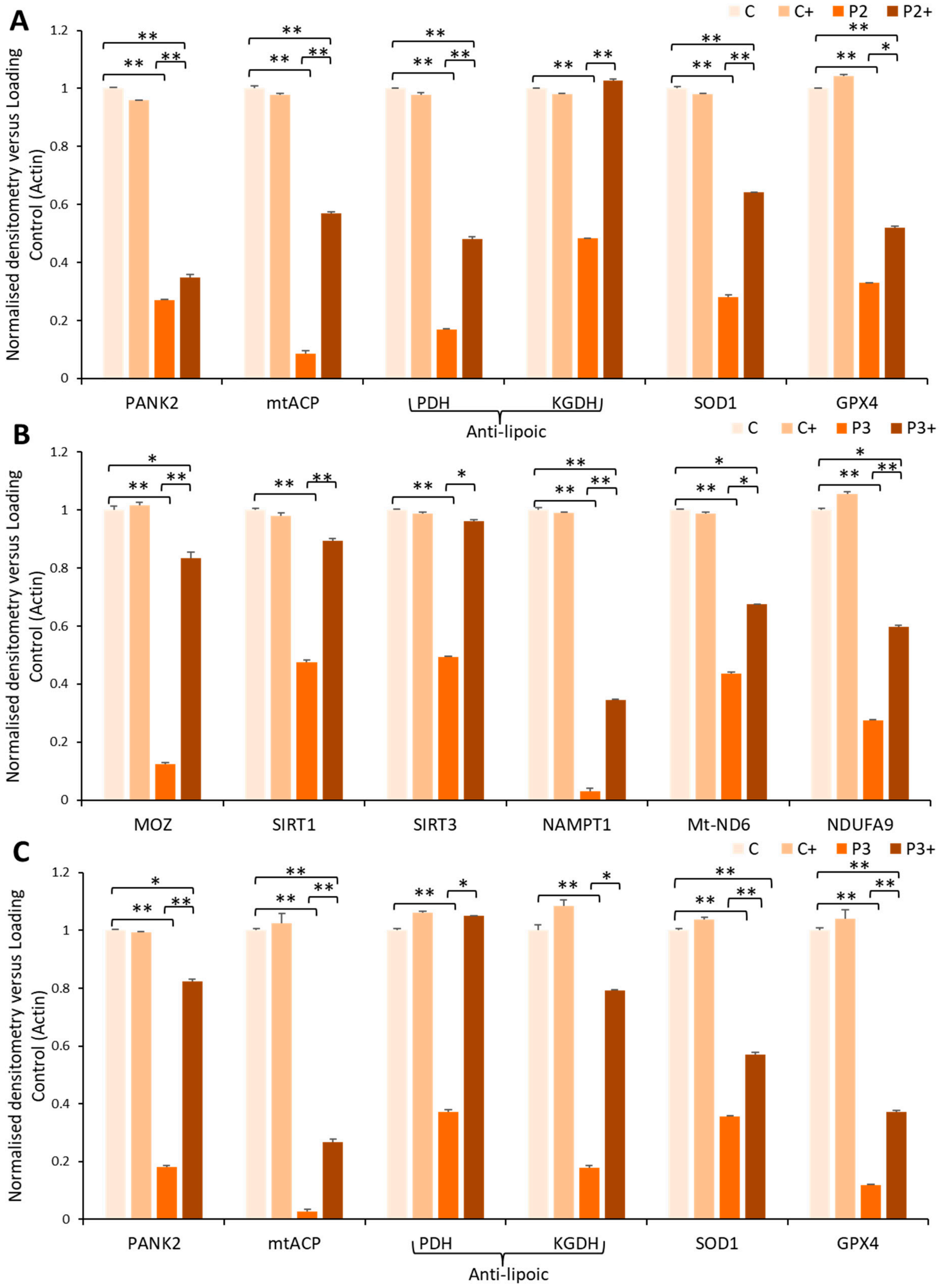

Supplementary Figure S8. Western blotting quantification of figure 3. Patients in figure panels: patient 2 (P2) (A) and patient 3 (P3) (B-C). Data were normalized to actin. Data represent the mean ±SD of three separate experiments. *p-value < 0.05 and **p-value < 0.01.

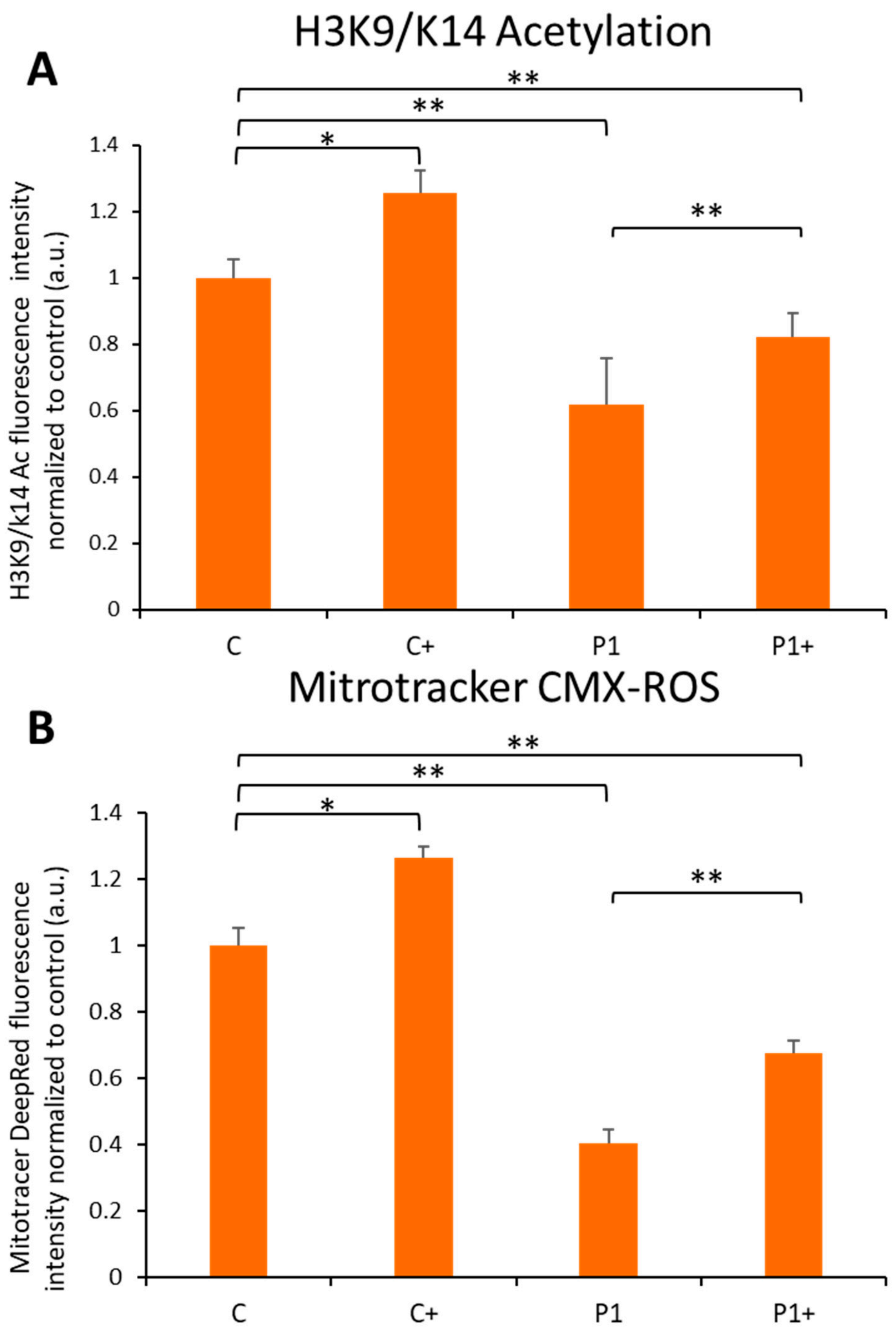

Supplementary Figure S9. Quantification of H3K9/K14 Acetylation and MitoTracker staining of figure 4. It was used softWoRx and ImageJ software. Data were normalized to control. Fifty cells per condition were analyzed. Data represent the mean ±SD of three separate experiments. *p-value < 0.05 and **p-value < 0.01.

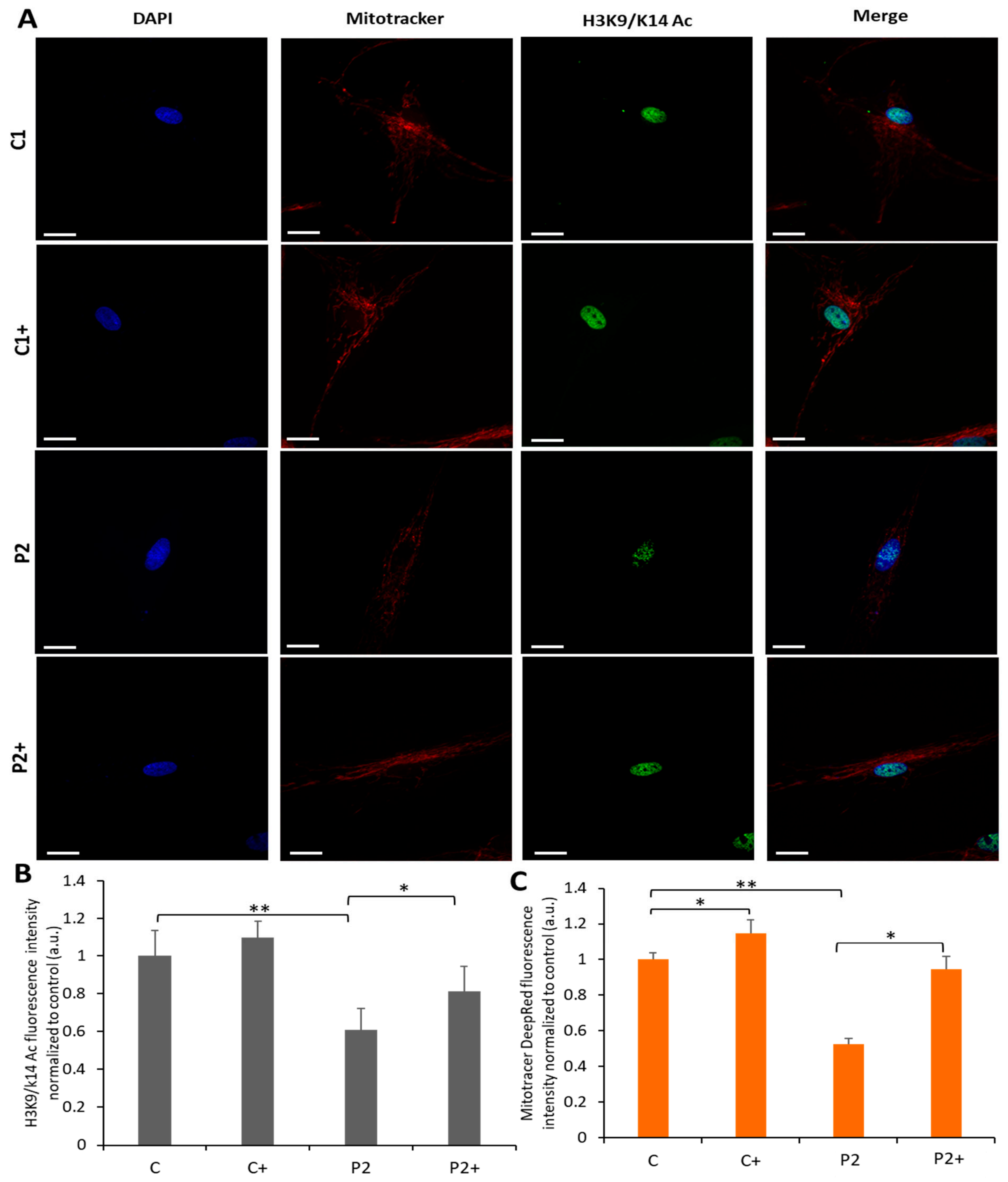

Supplementary Figure S10. Pantothenate and L-carnitine treatment increases histone acetylation levels and mitochondrial membrane potential in KAT6A fibroblasts. Control (C) and mutant P2 fibroblasts (Patient 2, P2) were treated with 0.4 µM pantothenate and 0.4 µM L-carnitine for 15 days (C+ and P2+). (A) Control and KAT6A fibroblasts were incubated with Mitotracker CMX-ROS FM 100 nM for 45 min, then they were fixed and immunostained with anti-H3K9/K14 and examined by fluorescence microscopy. (B) Quantification of H3K9/K14 Acetylation immunostaining. (C) Quantification of MitoTracker staining. Fifty cells per condition were analyzed. Fluorescence was quantified using softWoRx and ImageJ software. Data represent the mean ±SD of three separate experiments. *p-value < 0.05 and **p-value < 0.01. Scale bar= 15 µm.

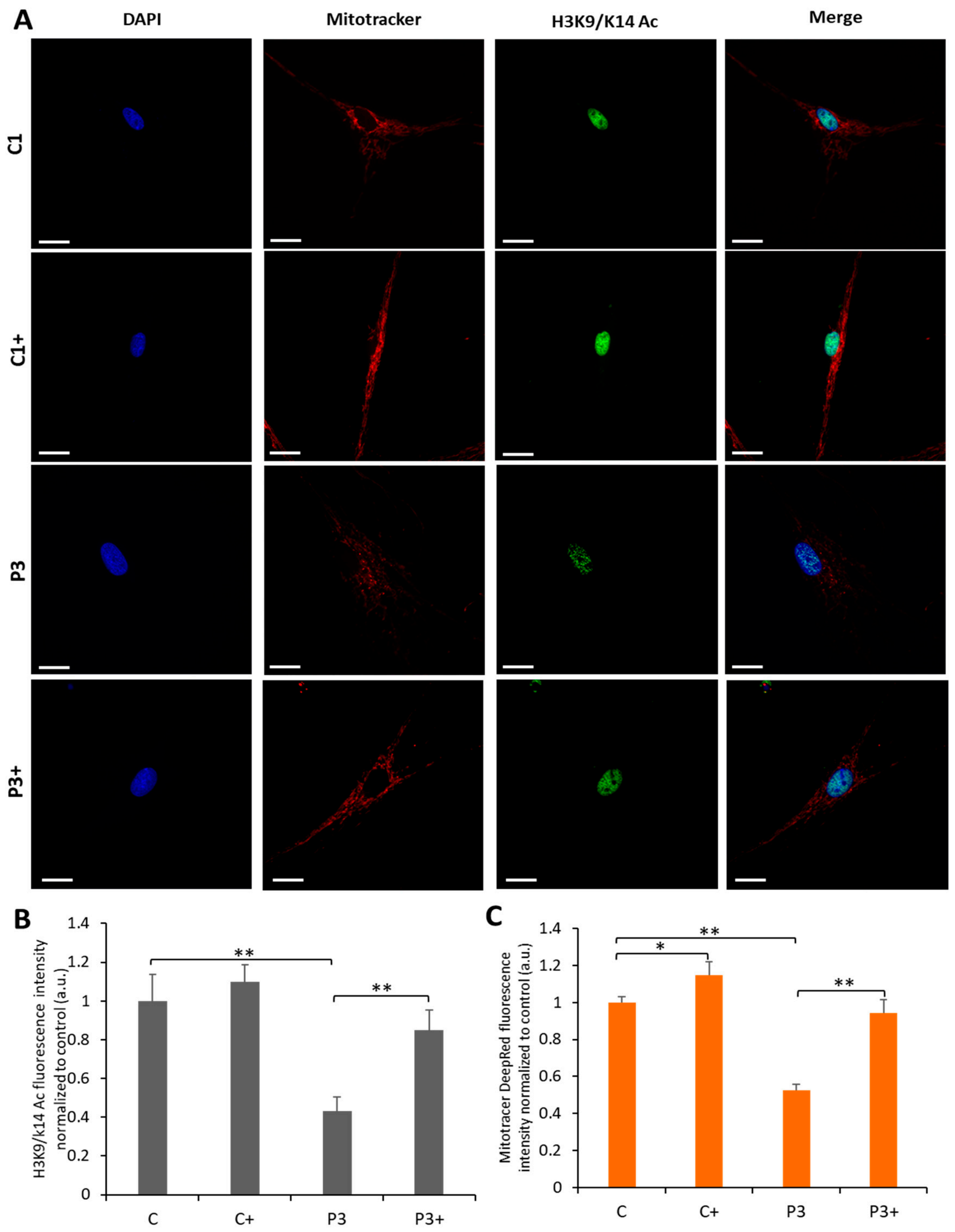

Supplementary Figure S11. Pantothenate and L-carnitine treatment increases histone acetylation levels and mitochondrial membrane potential in KAT6A fibroblasts. Control (C) and mutant P3 fibroblasts (Patient 3, P3) were treated with 0.7 µM pantothenate and 0.7 µM L-carnitine for 15 days (C+ and P3+). (A) Control and KAT6A fibroblasts were incubated with Mitotracker CMX-ROS FM 100 nM for 45 min, then they were fixed and immunostained with anti-H3K9/K14 and examined by fluorescence microscopy. (B) Quantification of H3K9/K14 Acetylation immunostaining. (C) Quantification of MitoTracker staining. Fifty cells per condition were analyzed. Fluorescence was quantified using softWoRx and ImageJ software. Data represent the mean ±SD of three separate experiments. *p-value < 0.05 and **p-value < 0.01. Scale bar= 15 µm.

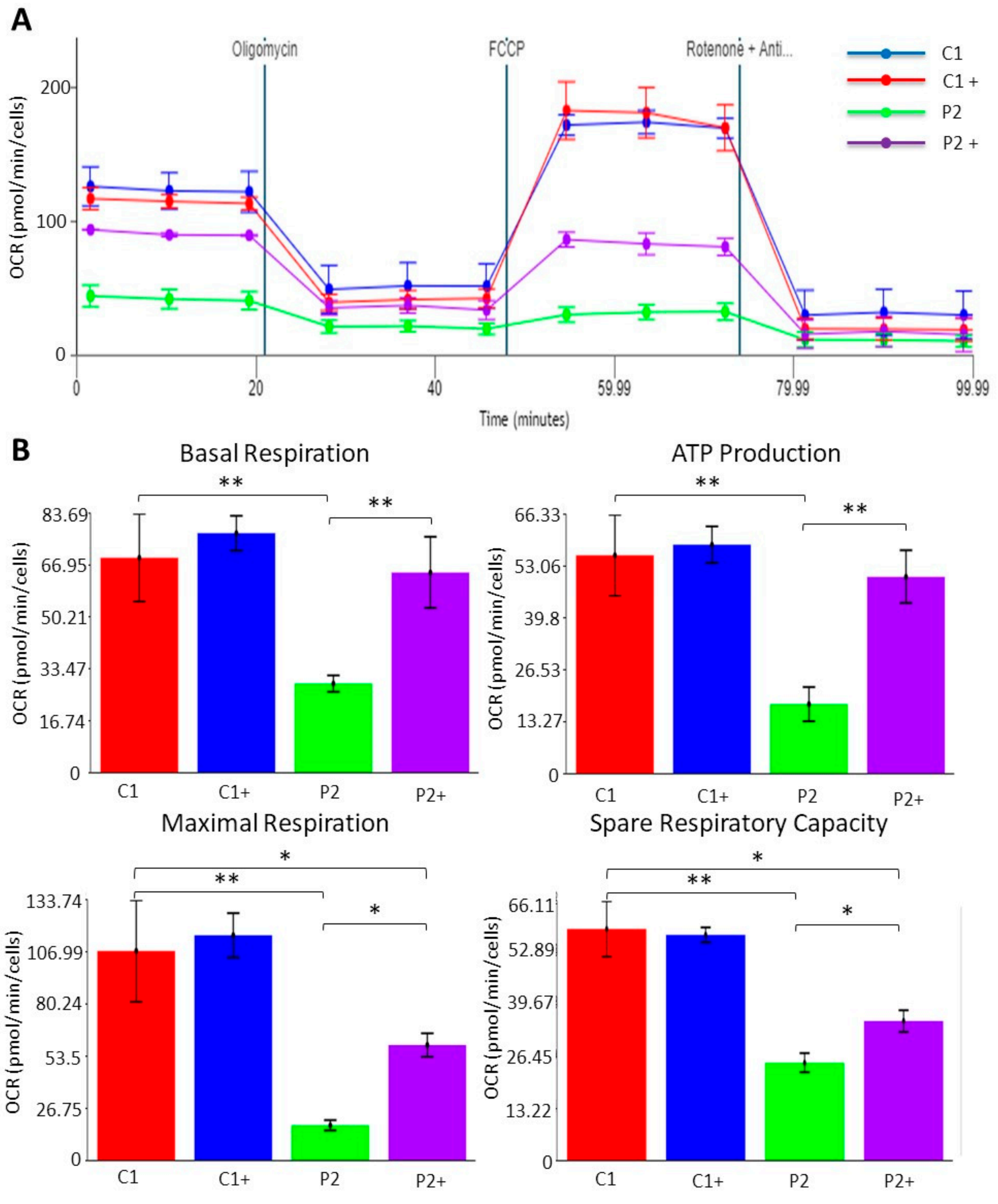

Supplementary Figure S12. Pantothenate and L-carnitine supplementation improves cell bioenergetics of mutant KAT6A fibroblasts. Control (C) and KAT6A fibroblasts (Patient 2, P2) were treated for 15 days with 0.4 μM pantothenate and 0.4 μM L-carnitine (C+ and P2+). (A) Mitochondrial respiration profile was measured with a Seahorse XFe24 analyzer. (B) Basal respiration, ATP production, maximal respiration and spare respiratory capacity were assessed by Seahorse analytics website. *p-value < 0.05 and **p-value < 0.01.

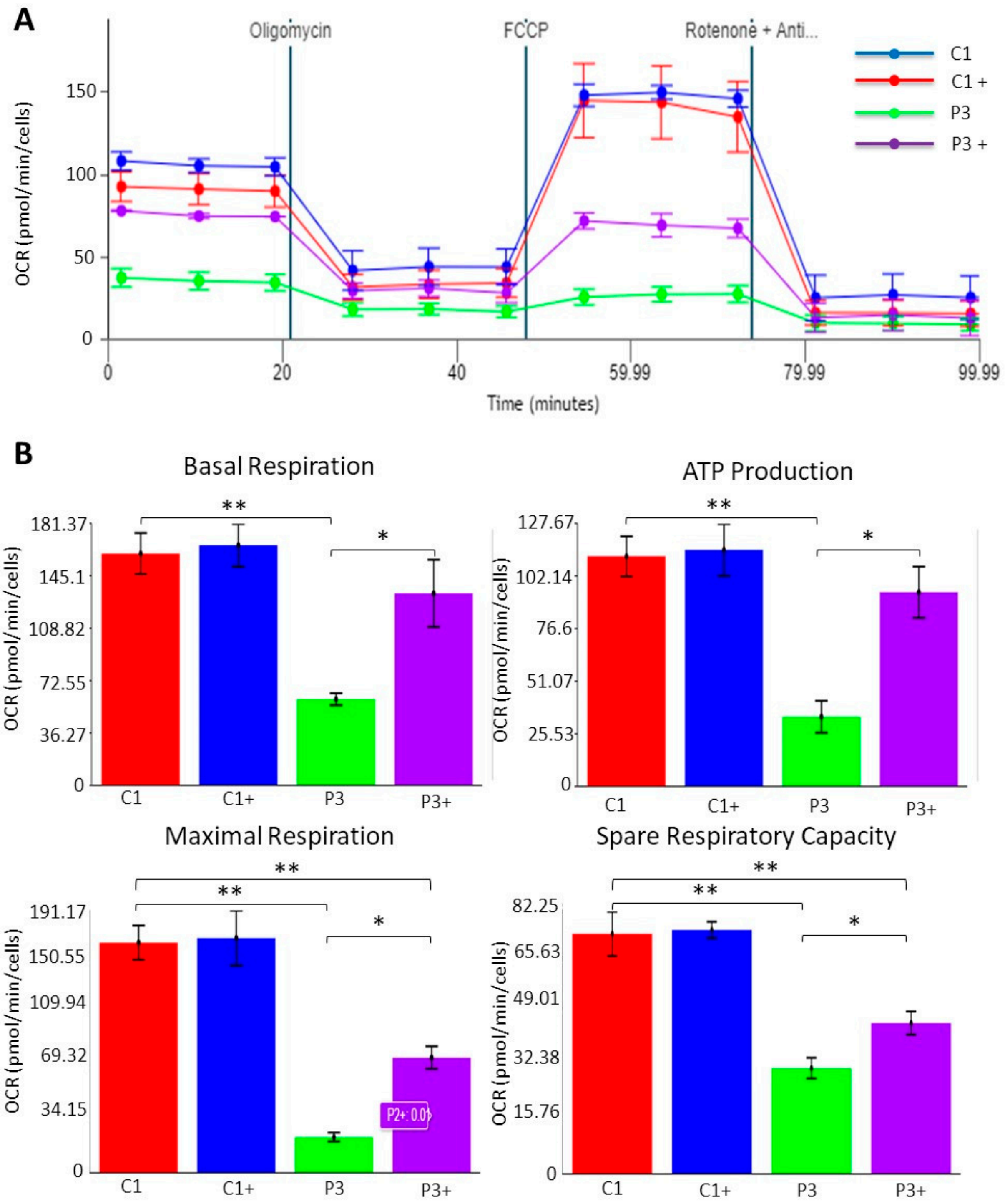

Supplementary Figure S13. Pantothenate and L-carnitine supplementation improves cell bioenergetics of mutant KAT6A fibroblasts. Control (C) and KAT6A fibroblasts (Patient 3, P3) were treated for 15 days with 0.7 µM pantothenate and 0.7 µM L-carnitine (C+ and P3+). (A) Mitochondrial respiration profile was measured with a Seahorse XFe24 analyzer. (B) Basal respiration, ATP production, maximal respiration and spare respiratory capacity were assessed by Seahorse analytics website. *p-value < 0.05 and **p-value < 0.01.

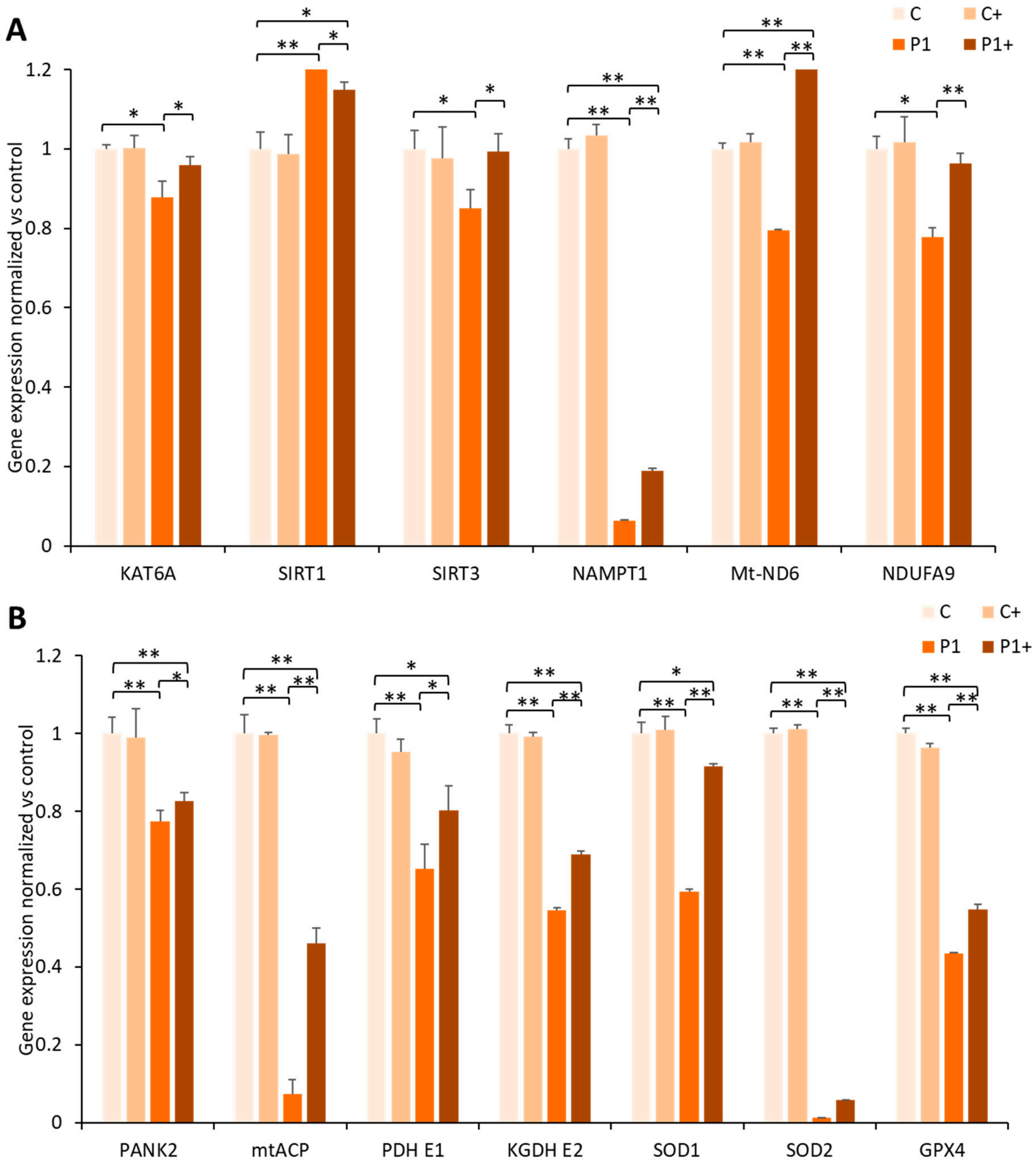

Supplementary Figure S14. Transcript expression levels of key altered genes in control (C) and mutant KAT6A fibroblasts (P1) with (+) and without pantothenate and L-carnitine treatment. Gene expression was retrieved from the DESeq2 RNAseq differential expression analysis. DESeq2 provides methods to test for differential expression by using negative binomial generalized linear models. Data were normalized versus control. Data represent the mean ±SD of three separate experiments. *p-value < 0.05 and **p-value < 0.01. RNAseq data are available in a supplementary file (RNAseq-data.xlsx).

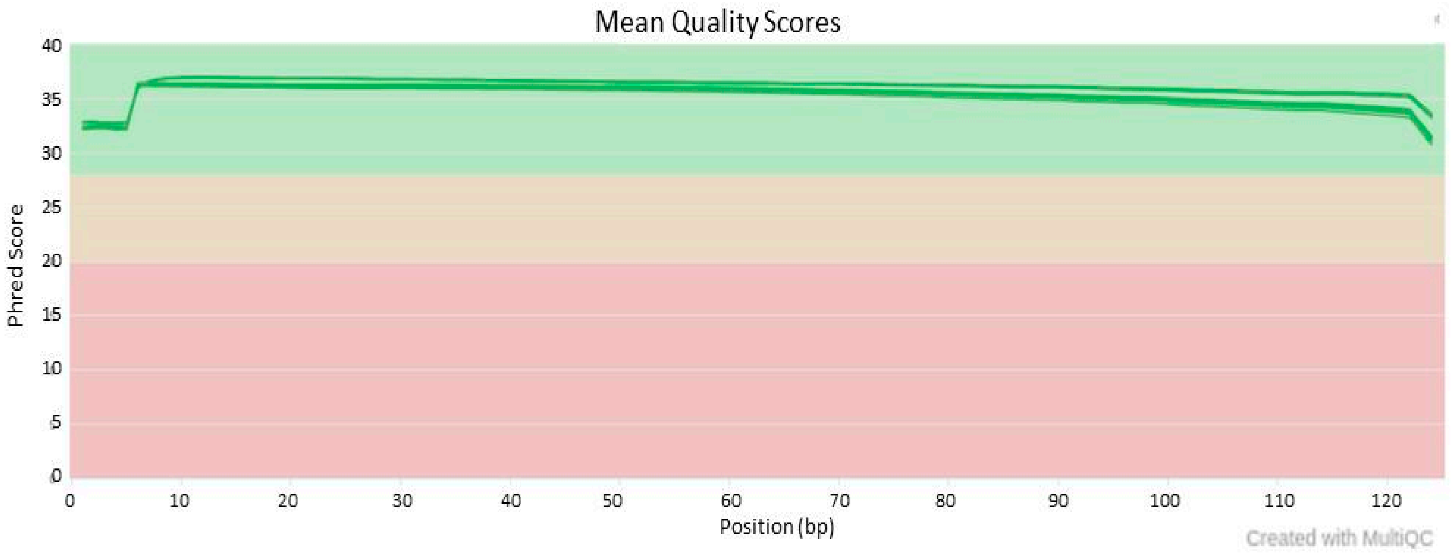

Supplementary Figure S15. Quality control in RNAseq. Quality control in control and mutant KAT6A fibroblasts treated and non-treated with a Phred-Score (Q) greater than 30.
